# Increased pro-inflammatory cytokines, glial activation and oxidative stress in the hippocampus after short-term bilateral adrenalectomy

**DOI:** 10.1186/s12868-016-0296-1

**Published:** 2016-09-01

**Authors:** Naserddine Hamadi, Azimullah Sheikh, Nather Madjid, Loai Lubbad, Naheed Amir, Safa Al-Deen Saudi Shehab, Fatima Khelifi-Touhami, Abdu Adem

**Affiliations:** 1 Department of Pharmacology, College of Medicine and Health Science, United Arab Emirates University, 17666 Maqam, Al Ain, United Arab Emirates; 2 Department of Neuroscience, Karolinska Institutet, 171 77 Stockholm, Sweden; 3 Department of Surgery, College of Medicine and Health Science, United Arab Emirates University, 17666 Maqam, Al Ain, United Arab Emirates; 4 Department of Anatomy, College of Medicine and Health Science, United Arab Emirates University, 17666 Maqam, Al Ain, United Arab Emirates; 5 Ethnobotany-Palynology and Ethnopharmacology-Toxicology Laboratory, Department of Animal Biology, Constantine-1 University, 25000 Constantine, Algeria

**Keywords:** Adrenalectomy, Hippocampus, Neuroinflammation, Neurodegeneration, Oxidative stress

## Abstract

**Background:**

Bilateral adrenalectomy has been shown to damage the hippocampal neurons. Although the effects of long-term adrenalectomy have been studied extensively there are few publications on the effects of short-term adrenalectomy. In the present study we aimed to investigate the effects of short-term bilateral adrenalectomy on the levels of pro-inflammatory cytokines IL-1β, IL-6 and TNF-α; the response of microglia and astrocytes to neuronal cell death as well as oxidative stress markers GSH, SOD and MDA over the course of time (4 h, 24 h, 3 days, 1 week and 2 weeks) in the hippocampus of Wistar rats.

**Results:**

Our results showed a transient significant elevation of pro-inflammatory cytokines IL-1β and IL-6 from 4 h to 3 days in the adrenalectomized compared to sham operated rats. After 1 week, the elevation of both cytokines returns to the sham levels. Surprisingly, TNF-α levels were significantly elevated at 4 h only in adrenalectomized compared to sham operated rats. The occurrence of neuronal cell death in the hippocampus following adrenalectomy was confirmed by Fluoro-Jade B staining. Our results showed a time dependent increase in degenerated neurons in the dorsal blade of the dentate gyrus from 3 days to 2 weeks after adrenalectomy. Our results revealed an early activation of microglia on day three whereas activation of astroglia in the hippocampus was observed at 1 week postoperatively. A progression of microglia and astroglia activation all over the dentate gyrus and their appearance for the first time in CA3 of adrenalectomized rats hippocampi compared to sham operated was seen after 2 weeks of surgery. Quantitative analysis revealed a significant increase in the number of microglia (3, 7 and 14 days) and astrocytes (7 and 14 days) of ADX compared to sham operated rats. Our study revealed no major signs of oxidative stress until 2 weeks after adrenalectomy when a significant decrease of GSH levels and SOD activity as well as an increase in MDA levels were found in adrenalectomized compared to sham rats.

**Conclusion:**

Our study showed an early increase in the pro-inflammatory cytokines followed by neurodegeneration and activation of glial cells as well as oxidative stress. Taking these findings together it could be speculated that the early inflammatory components might contribute to the initiation of the biological cascade responsible for subsequent neuronal death in the current neurodegenerative animal model. These findings suggest that inflammatory mechanisms precede neurodegeneration and glial activation.

## Background

Glucocorticoids are steroid hormones produced in the adrenal gland and secreted in the blood stream in response to the circadian rhythm as well as stress situations [1]. The human and rodent versions of these hormones are cortisol and corticosterone respectively. It is well known since the second half of last century that the hippocampus is the main target of these hormones due to the high level of their receptors [2]. In addition, several other brain areas such as the amygdala, neocortex, hypothalamus are targets of the adrenal hormones because they contain mineralo and or glucocorticoid receptors [3].

Immunohistochemical studies showed glucocorticoid receptors in the brain are of two types, mineralocorticoid or type 1 and glucocorticoid or type 2 receptors [4]. The abundance of these receptors in the hippocampus differs from one neuronal population to another where the granule cells are rich in type 1 and pyramidal neurons in type 2 receptors. In the brain such hormones, in addition to their suppressive effect of inflammatory mediators [5], they play a major role in the production of neurotrophin-3 (NT-3), Brain Derived Growth factor (BDNF), Nerve Growth Factor (NGF) [6–9].

Several studies showed dual effect of these hormones on the hippocampus. It has been reported chronic administration of high dose of glucocorticoids results in degeneration of pyramidal neurons [10]. However, multiple studies have demonstrated that adrenalectomy (ADX) induced massive and selective degeneration of the granule cells of the dentate gyrus [11–13]. Moreover, in addition to granule cells degeneration, pyramidal cells degeneration were observed in the hippocampus after long-term adrenalectomy.

The discovery by Sloviter et al. [11] that adrenalectomy induces hippocampal granule cell loss and the duplication of this finding by several groups [7, 14–16] as well as the findings by Sapolsky et al. [17] and Adem et al. [18] that showed hippocampal pyramidal cell loss after long-term adrenalectomy have established this model as a neurodegenerative model of hippocampal cells. Moreover, the neurodegeneration and glial response to the cell death that takes place in the this model is comparable to what is seen in other established neurodegenerative models such ischemia [19, 20], surgical injury [21], Trimethyltin (TMT) [22] KA (kainic acid) [23] and cis-2,4-methanoglutamate (MGlu) [24]. Hence the ADX model can be used as an experimentally controlled neuronal cell death in the hippocampus which might be a relevant model to what is happening in neurodegenerative disorders.

Pro-inflammatory cytokines interleukin-1β (IL-1β) [25–27], Interleukin-6 (IL-6) [28, 29] and tumor necrosis factor alpha (TNF-α) [27, 30, 31] have been shown to be produced by different cells in the brain [19]. Elevated levels of such cytokines have been found in numerous degenerative diseases such as Alzheimer’s disease (AD) [32, 33], Amyotrophic lateral sclerosis (ALS) [25] and Parkinson’s disease (PD) [34, 35]. Different studies showed the early expression of cytokines in various neurodegenerative animal models in which kainic acid [36], TMT [37], ischemia–reperfusion [38, 39], 1-methyl-4-phenyl-1,2,3,6-tetrahydropyridine (MPTP) [40, 41] treatments were used. It has been reported that these inflammatory mediators are involved in initiation of the morphological changes which occur during neuronal death and exacerbation of the damage induced by different insults [42–47].

Oxidative stress is well-known as a cytotoxic phenomenon when imbalance between the antioxidants and oxidants occurs leading to different aspects of tissue endangerment and subsequently contribution to the neurodegenerative process [48–51]. Reactive oxygen species (ROS) have important physiological functions [52, 53]. Production of high levels of ROS can cause oxidative stress. Since oxidative stress can induce cell damage and promote inflammation [54], cells have a battery of anti-oxidizing molecules and enzymes to prevent the accumulation of ROS [55].

The aim of this study was to examine the impact of short-term bilateral adrenalectomy on production of pro-inflammatory cytokines, the activation of different glial cells, oxidative stress and the possibility of their association with neurodegeneration.

## Methods

### Animals and treatment

Eight-week old male Wistar rats 170–220 g were obtained from College of Medicine and Health Sciences animal facility (Al Ain, UAE) and used in the current study. Under Pentobarbital (35 mg/kg body weight) anesthesia, rats were subjected either to adrenalectomy or to sham operation (laparotomy) as described by Sloviter et al. [11], the naive animals were left untouched. Age matched naïve control groups were also used. All animals were placed in Plexiglas cages; four rats per group, maintained in a temperature (22 °C) and 12:12 light–dark cycle conditions, animals received ad libitum access to food and water throughout the experiment. Adrenalectomized rats were provided with 0.9 % saline in place of drinking water in order to maintain electrolyte balance and prevent the deleterious effects of sodium chloride insufficiency. The regional ethical committee of the College of Medicine and Health Sciences (UAE University) approved this study (RECA/01/05).

### Determination of plasma corticosterone levels

The levels of corticosterone in the serum were used to assess the efficacy of adrenalectomy, approximately 1 ml blood samples were taken directly from the heart at the time of sacrifice. For both biochemical and histological assessments (n = 120) rats were sacrificed by decapitation after surgery at different time points: 4 h (n = 24), 24 h (n = 24), 3 days (n = 24), 1 week (n = 24) and 2 weeks (n = 24). The sera samples were stored at −80 °C until corticosterone levels were measured by Enzyme Immunoassay (EIA) Kit (Life sciences, Lausen, Switzerland). The assay is based on the competitive binding technique as described by the manufacturer. Briefly, a polyclonal antibody specific for corticosterone becomes bound to the donkey anti-sheep antibody coated onto the micro-plate. Following a wash to remove excess polyclonal antibody, corticosterone present in the serum competes with a fixed amount of horseradish peroxidase (HRP)-labeled corticosterone for sites on the polyclonal antibody. This is followed by another wash to remove excess of the conjugate and unbound sample. A substrate solution is added to the wells to determine the bound enzyme activity. The color development is stopped and the absorbance is read at 450 nm. The intensity of the color is inversely proportional to the concentration of corticosterone in the sample. A standard curve in the range 32–20,000 pg/ml was constructed to calculate corticosterone concentrations in the samples.

### Enzyme-linked immunosorbent assay (ELISA)

Animals (n = 80) were sacrificed by decapitation after surgery at different times: 4 h (Sham = 8, ADX = 8), 24 h (Sham = 8, ADX = 8), 3 days (Sham = 8, ADX = 8), 1 week (Sham = 8, ADX = 8) and 2 weeks (Sham = 8, ADX = 8). Similarly naïve controls (n = 8) were sacrificed by decapitation. The brain was removed, the hippocampus was immediately dissected out on ice, frozen in liquid nitrogen and stored at −80 °C until assayed. Thawed hippocampal tissues were homogenized in ice-cold T-PER tissue extraction buffer (Pierce Biotechnology, Rockford, IL,USA), supplemented with 1:100 protease inhibitor cocktail (Sigma, Saint Louis, MO, USA). After sonication, samples were centrifuged at 16,000 rpm for 30 min at 4 °C and the supernatant was collected and stored at −80 °C until use. Protein concentrations were determined using Bradford’s protein assay kit (Bio-Rad, Stockholm, Sweden) and levels of cytokines (IL-1β, IL-6, TNF-α) were assessed by ELISA using the commercially provided antibodies and standard proteins (R&D Systems; Minneapolis, USA).

Briefly, standard 96-well flat-bottom NUNC-Immunomaxisorp ELISA plates (NUNC, Roskilde, Denmark) were incubated at room temperature (RT) overnight with the captured antibody, which binds the cytokine of interest. The next day the plates were blocked by incubation for 2 h at RT with 1 % bovine serum albumin (Sigma, Saint Louis, MO, USA) in PBS. Serial dilutions of known amounts of standard protein were applied in duplicate for the standard curve. The plates containing the standard proteins and supernatants of hippocampal tissue homogenates were incubated at RT for overnight.

The following day, the plates were incubated with the corresponding biotinylated detecting antibody for 1 h at RT. An avidinylated antibody conjugated to horseradish peroxidase (HRP) as a tertiary reactant was incubated for 20 min at RT. Tetramethylbenzidine (41.6 mM) at 100 μl per well was used to develop color. The reaction was terminated with 2 M sulfuric acid and the absorbance was recorded at 450 nm in a plate reader within 10 min after stopping of the reaction. The levels of IL-1β, IL-6 and TNF-α were evaluated by comparison with the regression curve for each cytokine standard. Calculated cytokine concentrations were normalized to the total protein concentrations obtained in the Bradford’s protein assay for each sample. Cytokines concentration is reported as pg of cytokine/mg of protein.

### FJB staining

Fluoro-Jade B staining was performed as described by Schmued and Hopkins [56]. 32 animals, 4 h (Sham = 4, ADX = 4), 24 h (Sham = 4, ADX = 4), 3 days (Sham = 4, ADX = 4), 1 week (Sham = 4, ADX = 4) and 2 weeks (Sham = 4, ADX = 4) were used for this staining. The coronal sections of the brain were first deparaffinized through two changes of xylene each 10 min and then rehydrated through a graduated alcohol series. The slides were immersed in 1 % sodium hydroxide in 80 % alcohol (20 ml of 5 % NaOH added to 80 ml absolute alcohol) for 5 min. The slides are then kept in 70 % alcohol and in distilled water for 2 min respectively. The slides were then transferred to a solution of 0.06 % potassium permanganate for 10 min. The slides were then rinsed in distilled water for two min and immersed in 0.0004 % Fluoro-Jade B (Darmstadt, Germany) for 20 min. The slides were then rinsed for one min in each of three distilled water washes and placed on a slide warmer for 10 min. The dry slides were cleared by immersion in xylene for at least 1 min and were then cover slipped with DPX.

### Immunoflurescent labeling

Animals (n = 32) were sacrificed after surgery at different times: 4 h (Sham = 4, ADX = 4), 24 h (Sham = 4, ADX = 4), 3 days (Sham = 4, ADX = 4), 1 week (Sham = 4, ADX = 4) and 2 weeks (Sham = 4, ADX = 4). Animals were deeply anesthetized with intra-peritoneal administration of sodium pentobarbital (35 mg/kg body weight) and perfused transcardially through the ascending aorta, with 50 ml of 0.1 M phosphate-buffered saline followed by 300 ml of freshly prepared solution of 4 % formaldehyde in 0.1 M phosphate buffer. Brains were removed and post fixed for 1 week in the same fixative at 4 °C. All tissues were then dehydrated through an ethanol series, cleared in 100 % xylene and embedded in paraffin. A 1 μm coronal sections (n = 10 per animal) of the brain were selected approximately −3.7 mm relative to bregma according to Paxinos and Watson [57] and mounted on gelatin subbed slides.

For all fluorescence labeling, the paraffin-embedded tissue sections were deparaffinized with xylene, rehydrated with ethanol (100, 90, 80, and 70 %) and finally washed in distilled water. To unmask the epitopes and obtain efficient immunostaining, the brain sections were subjected to an antigen retrieval procedure with 10 mM Sodium Citrate (pH 6) followed by microwave heating for 1 min at high power and 10 min at low power. After this procedure the samples were washed with 0.1 M phosphate buffered saline (PBS, pH 7.4) and cooled down at room temperature.

For double-immunofluorescence labeling, the sections were blocked by 1 % BSA at room temperature for 30 min and then were incubated with each primary antibody at 4 °C overnight. The primary antibodies used were rabbit polyclonal anti-ionized calcium-binding adaptor molecule 1 (Iba1, Wako, MA, USA 1:2000) mixed with mouse monoclonal anti-neuronal nuclear antigen antibody (Neun, Millipore, MA, USA, 1:1000) and rabbit polyclonal anti-glial fibrillary acidic protein (GFAP, Dako, Copenhagen, Denmark, 1:1000) mixed with mouse monoclonal anti-neuronal nuclear antigen (Neun, Millipore, MA, USA, 1:1000). After washing three times in PBS, tissue sections were incubated with donkey anti-rabbit conjugated to Alexa 488 (Invitrogen, Paisley, UK, 1:200) mixed with donkey anti-mouse conjugated to Rhodamine (Jakson, Pennsylvania, USA, 1:100) all diluted in PBS-Triton X-100 0.3 % for 1 h at room temperature. Afterwards, the sections were washed several times in PBS; slides were cover slipped with fluoromount medium.

Similar procedure was used for triple-immunofluorescence labeling in order to define the localization of microglia, astroglia and neurons in the same brain section, the mixture of the following primary antibodies was used, rabbit GFAP (Dako, Copenhagen, Denmark, 1:1000) mixed with mouse Neun (Millipore, MA, USA, 1:1000) and goat Iba1 (Abcam, MA, USA 1:1000) at 4 °C overnight. Regarding the secondary antibody, tissue sections were incubated with donkey anti-rabbit conjugated to Alexa 488 (Invitrogen, Paisley, UK, 1:200) mixed with donkey anti-mouse conjugated to Rhodamine (Jakson, Pennsylvania, USA, 1:100) and donkey anti-goat conjugated to cy5 (Jakson, Pennsylvania, USA, 1:100).

Representative digital images were captured using a Zeiss AxioCamHRc Digital camera with AxioVision 3.0 software (Carl Zeiss, Germany). Some sections were also examined with a C1 laser scanning confocal microscope (Nikon, Tokyo, Japan). 4× and 20× objectives were used to capture confocal images for the preparation of Figs. 3, 5 and 7 respectively where only one optical section was utilized. The resulting files were used to generate the figures in Adobe PhotoShopCS6 where the photomicrographs were adjusted for contrast and brightness in order to determine the relationship between labeled neurons, astrocytes and microglia.

### Quantitative analysis of the Iba-l and GFAP positive cells

Both microglia and astrocytes undergo morphological changes and became reactive cells in response to CNS injury. In this study we investigated the response of both these glial cells in the hippocampus after ADX. Typically, reactive microglia show shortening and widening of their processes and some of them they show amoeboid shape by losing their processes. Reactive astrocytes show increased size of their processes. These features can be easily visualized in sections stained with Iba1 and GFAP antibodies as markers for microglia and astrocytes respectively (Figs. 3, 5). In this study, only positively stained Iba1-positive microglia and GFAP-positive astrocytes with visible cell body at the focal plane were counted. Three ADX and three sham rats were used for each time point. Three sections of the hippocampus separated by 30 µm were used from each rat. From each section three optical fields were selected for cell counting. Images of stained sections were taken at 20× magnification using an AxioCam HRc Digital camera with AxioVision 3.0 software (Carl Zeiss, Germany). In order to avoid being biased, the parameters of acquiring images like the time of exposure and the gain were first fixed for labeled microglia and astroglia and kept constant for all the examined time points. Cells matching the above mentioned criteria were counted manually using cell counter software (Heracle BioSoft SRL, Romania). A comparison was then carried out between the number of microglia and astrocytes in ADX and sham operated rats.

### Colorimetric assay

Animals (n = 80) were sacrificed by decapitation after surgery at different times: 4 h (Sham = 8, ADX = 8), 24 h (Sham = 8, ADX = 8), 3 days (Sham = 8, ADX = 8), 1 week (Sham = 8, ADX = 8) and 2 weeks (Sham = 8, ADX = 8). The brain was removed, the hippocampus was immediately dissected out on ice, frozen in liquid nitrogen and stored at −80 °C until use for the colorimetric assays to determine the levels of oxidative stress markers GSH, SOD and MDA.

The concentration of reduced glutathione (GSH) was determined in whole tissue supernatant. The hippocampal sample was first deproteinized with the 5 % of 5-sulfosalicylic acid solution followed by centrifugation to remove the precipitated protein and then assayed for glutathione using the commercial kit (Sigma, Saint Louis, MO, USA). The measurement of GSH uses a kinetic assay in which catalytic amounts (nmoles) of GSH cause a continuous reduction of 5, 5′-dithiobis (2-nitrobenzoic acid) (DTNB) to TNB and the GSSG formed is recycled by glutathione reductase and NADPH. The yellow product, 5-thio-2-nitrobenzoic acid (TNB) is measured spectrophotometrically at 412 nm. The assay uses a standard curve of reduced glutathione to determine the amount of glutathione in the sample [58].

SODs are metalloenzymes that catalyze the dismutation of the superoxide radical (O_2_^·−^) into hydrogen peroxide (H_2_O_2_) and molecular oxygen (O_2_). The assay kit utilizes a tetrazolium salt for detection of superoxide radicals generated by xanthine oxidase and hypoxanthine by using the commercial kit (Cayman Chemical, MI, and USA). One unit of SOD is defined as the amount of enzyme needed to exhibit 50 % dismutation of the superoxide radical. The plate was read at 440 nm using plate reader.

The measurement of the TBARS amount in the hippocampal homogenate is based on the interaction of thiobarbituric acid (TBA) with malondialdehyde (MDA) under acid conditions (acetic acid) and higher temperature (95 °C) (Cayman Chemical, MI, USA), the formed TBA-MDA adduct measured colorimetrically at 530–540 nm. The MDA level was evaluated by comparison with the regression curve for standard.

### Statistical analysis

All data are reported as the mean ± standard error of the mean and the analysis was considered significantly different if P ≤ 0.05. Results were analyzed by one way ANOVA for (IL-1β, IL-6 and TNF-α) and two-tail Student *t* test for (GSH, SOD and MDA) and microglia and astrocytes counting using SPSS version 20 (IBM, USA).

## Results

**Fig. 1:**
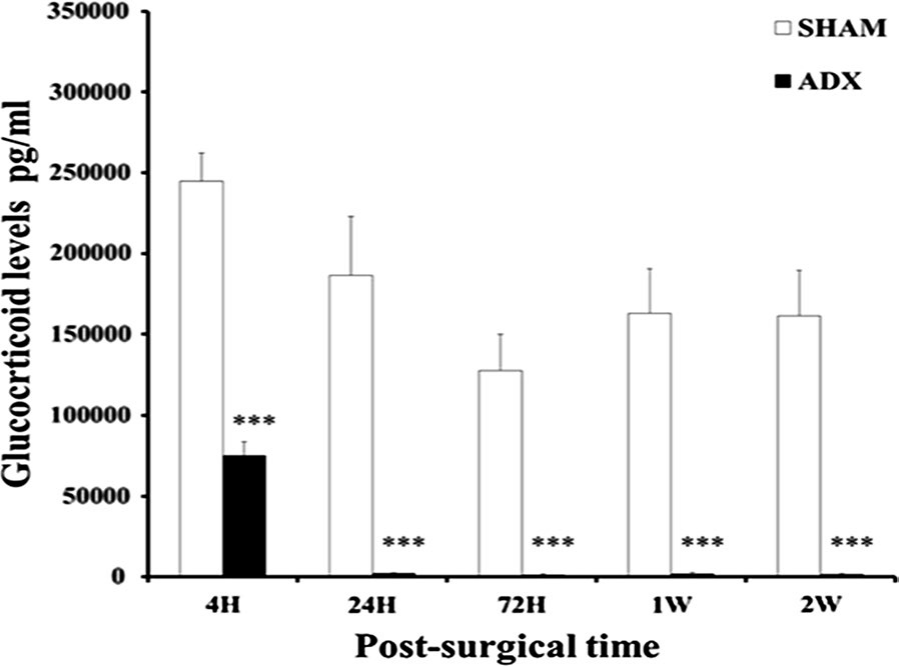
*Bar* graphs showing levels of plasma corticosterone. Levels of corticosterone were measured by EIA in the serum of adrenalectomized rats compared to the sham operated rats over the course of time (4 h, 24 h, 3 days, 1 week and 2 weeks). ***P < 0.001. Data are expressed as mean (±SEM)

Efficacy of adrenalectomy was confirmed post-mortem by analysis of plasma corticosterone levels. The levels of corticosterone were significantly decreased in the sera of adrenalectomized compared to bilateral sham operated rats after 4 h (P = 0.001), 24 h (P < 0.001), 72 h (P < 0.001), 1 week (P < 0.001), 2 weeks (P < 0.001) of surgery (Fig. 1).

### Increase in pro-inflammatory cytokines at early stages of adrenalectomy

The levels of pro-inflammatory cytokines IL-1β, IL-6 and TNF-α in the hippocampal homogenates of adrenalectomized, sham operated and naive rats were examined by ELISA over the course of time (4 h, 24 h, 3 days, 1 week and 2 weeks).

**Fig. 2:**
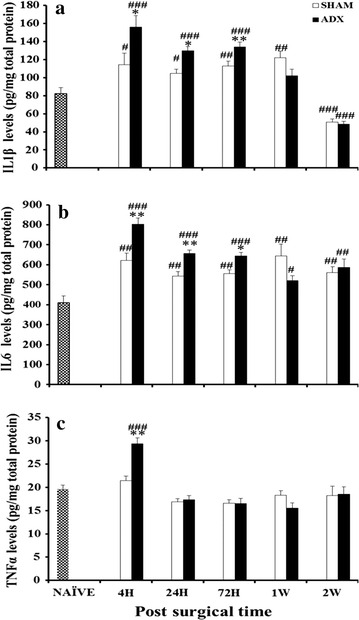
*Bar* graphs showing IL-1, IL-6 and TNF-α level in the hippocampus of adrenalectomized, sham operated and naive rats. Levels of IL-1 (**a**) IL-6 (**b**) and TNF-α (**c**) were measured by ELISA over the course of time (4 h, 24 h, 3 days, 1 week and 2 weeks). Sham versus ADX (*P < 0.05; **P < 0.01). Naive versus sham and ADX (^#^P < 0.05; ^##^P < 0.01; ^###^P < 001). Data are expressed as mean (±SEM)

The levels of IL-1β were significantly increased in the hippocampus of adrenalectomized rats compared to bilateral sham operated rats after 4 h (P < 0.05), 24 h (P < 0.05) and 3 days (P < 0.01) of surgery. However, one and 2 weeks after adrenalectomy no statistical difference in the hippocampal levels of IL-1β were found between the two groups. The comparison of both groups to the naive revealed that the levels of IL-1β were significantly increased in sham (P < 0.05) and ADX (P < 0.001) after 4 h, and 24 h, 3 days. After 1 week the statistical significance persisted only between the naïve and sham (P < 0.01). After 2 weeks the levels of IL-1β decreased significantly in both sham (P < 0.001) and ADX (P < 0.001) compared to the naïve rats group (Fig. 2a).

Regarding the pro-inflammatory cytokine IL-6, a significant increase in its levels was observed in the hippocampus 4 h (P < 0.01), 24 h (P < 0.01) and 3 days (P < 0.05) after adrenalectomy. However, no change was seen in IL-6 levels after 1 and 2 weeks postoperatively. The comparison of both groups to the naive showed that the levels of IL-6 was significantly increased in sham (P < 0.01) and ADX (P < 0.001) after 4 h, 24 h, and 3 days. After 1 week the statistical significance continued between sham (P < 0.01), ADX (P < 0.05) and the naïve. 2 weeks later there is still a significant high level of IL-6 in the sham (P < 0.01) and ADX (P < 0.01) groups compared to the naïve group (Fig. 2b).

The levels of TNF-α were significantly increased in the hippocampus of adrenalectomized rats compared to bilateral sham operated rats after 4 h only (P < 0.01). No statistical differences between the sham operated and naïve groups was seen over the course of time. However, a significant elevation of hippocampal TNF-α levels was only observed in the ADX (P < 0.001) compared to naïve rats after 4 h following the surgery (Fig. 2c).

### Neuronal cell death after adrenalectomy

In order to examine the occurrence of neuronal cell death in the hippocampus following adrenalectomy over a course of time (4 h, 24 h, 3 days, 1 week and 2 weeks), a Fluoro-Jade B staining was performed. FJB staining is a specific staining for neurons undergoing degeneration, regardless of the specific insult or mechanism of cell death.

**Fig. 3:**
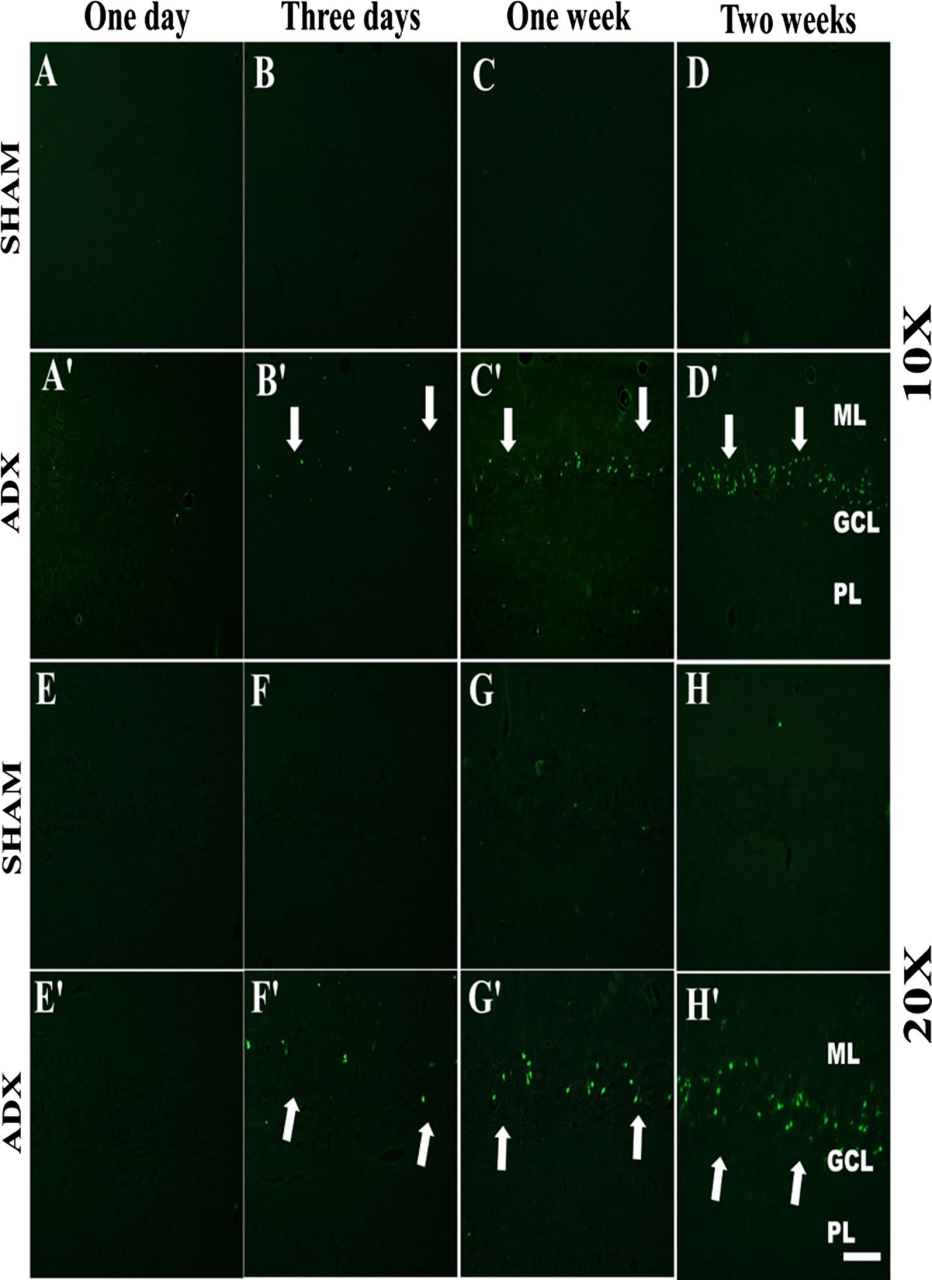
Images of coronal sections of the hippocampus stained with Fluoro-Jade B showing the progression of neuronal cell death over the course of time (1 day, 3 days, 1 week and 2 weeks) in the dentate gyrus of the hippocampus of adrenalectomized rats compared to sham operated rats. Absence of cell death was noticed in the dentate gyrus (**A**, **B**, **C**, **D**) and (**E**, **F**, **G**, **H**) of sham operated rats during the different time points (1 day, 3 days, 1 week and 2 weeks). 1 day after adrenalectomy the rats showed no signs of neuronal cell death in the dentate gyrus (**A**’) and (**E**’). A progression of neuronal cell death was seen in the dentate gyrus 3 days (**B**’), 1 week (**C**’), and 2 weeks (**D**’) after adrenalectomy. Molecular layer (ML), granule cell layer (GCL), polymorphous layers (PL). *Scale bar* 50 µm

**Fig. 4:**
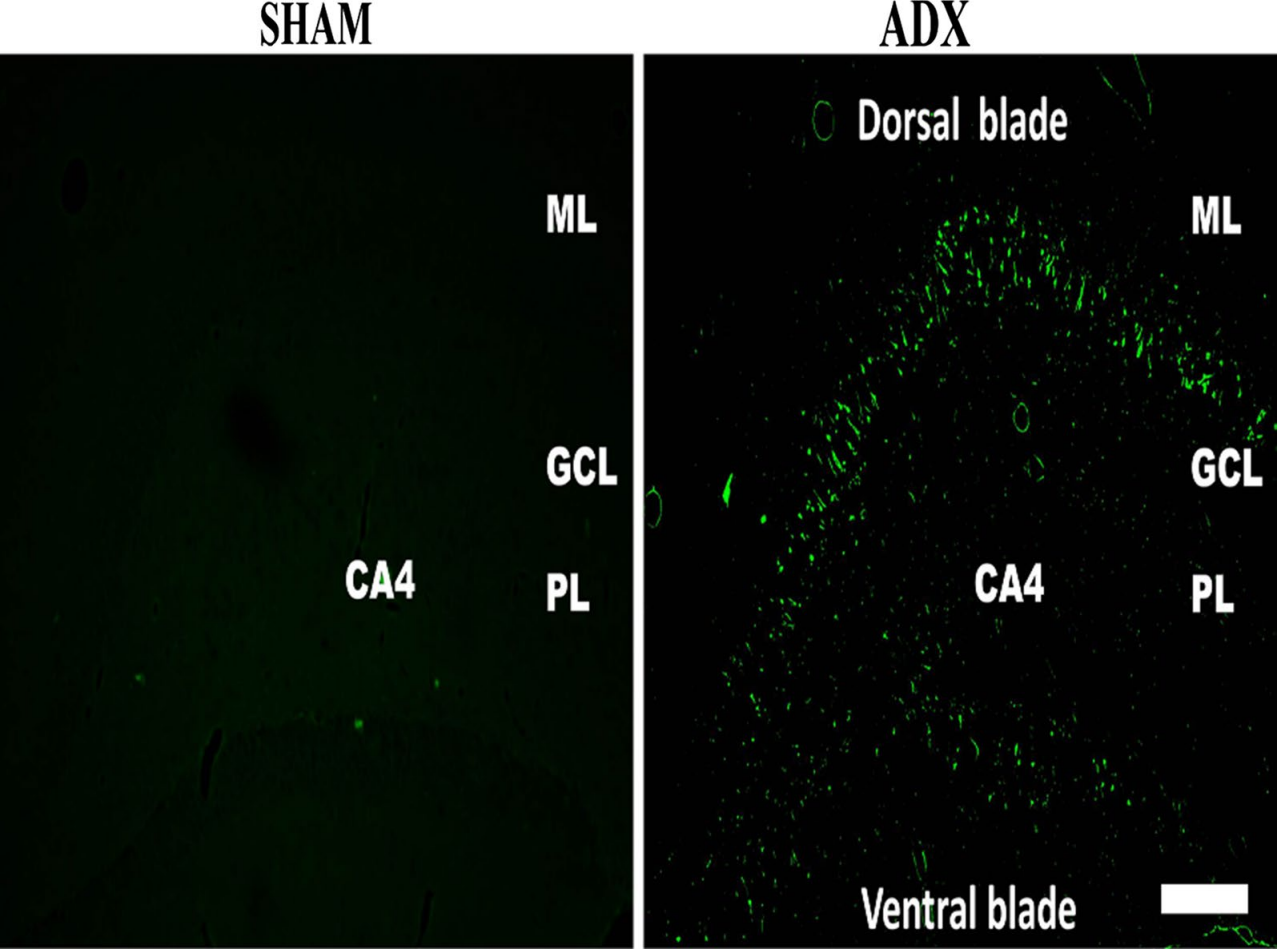
Representative confocal images of sections stained with Fluoro-Jade B (*green*) showing the occurrence of neurodegeneration after 2 weeks in both blades of the dentate gyrus of the hippocampus of adrenalectomized rats compared to sham operated rats. Molecular layer (ML), granule cell layer (GCL), polymorphous layers (PL). *Scale bar* 50 µm

After 4 h (results not shown) and 24 h following adrenalectomy our results showed no positive staining for degenerated neurons in both groups ADX and sham rats (Fig. 3A, A’, E, E’). 3 days after adrenalectomy we observed a few degenerated neurons in the dorsal blade of the dentate gyrus in the hippocampii of adrenalectomized rats only (Fig. 3B, B’, F, F’). Moreover, 1 week after adrenalectomy, more degenerated neurons were seen on the dorsal blade of the dentate gyrus (Fig. 3C’, G’) whilst no positive FJB staining was observed in sham operated rats (Fig. 3C, G). After 2 weeks of adrenalectomy, we observed a progression of cell death throughout the dorsal blade of the dentate gyrus (Fig. 3D’, H’) where an intense FJB staining was seen. In addition, less intense staining was observed in the ventral blade indicating that differential effect of the adrenal gland removal on both blades of the dentate gyrus. No positive FJB staining was observed in sham operated rats (Figs. 3D, H, 4).

### Adrenalectomy promotes activation of microglia and astroglia

The activation of microglia is one of the major signs of neurodegeneration. In order to investigate the activation of these cells in response to neurodegeneration occurring after short-term adrenalectomy, a double immunofluorescent labeling of neurons (Neun) and microglia (Iba-1) over the course of time (4 h, 24 h, 3 days, 1 week and 2 weeks) was performed.

**Fig. 5:**
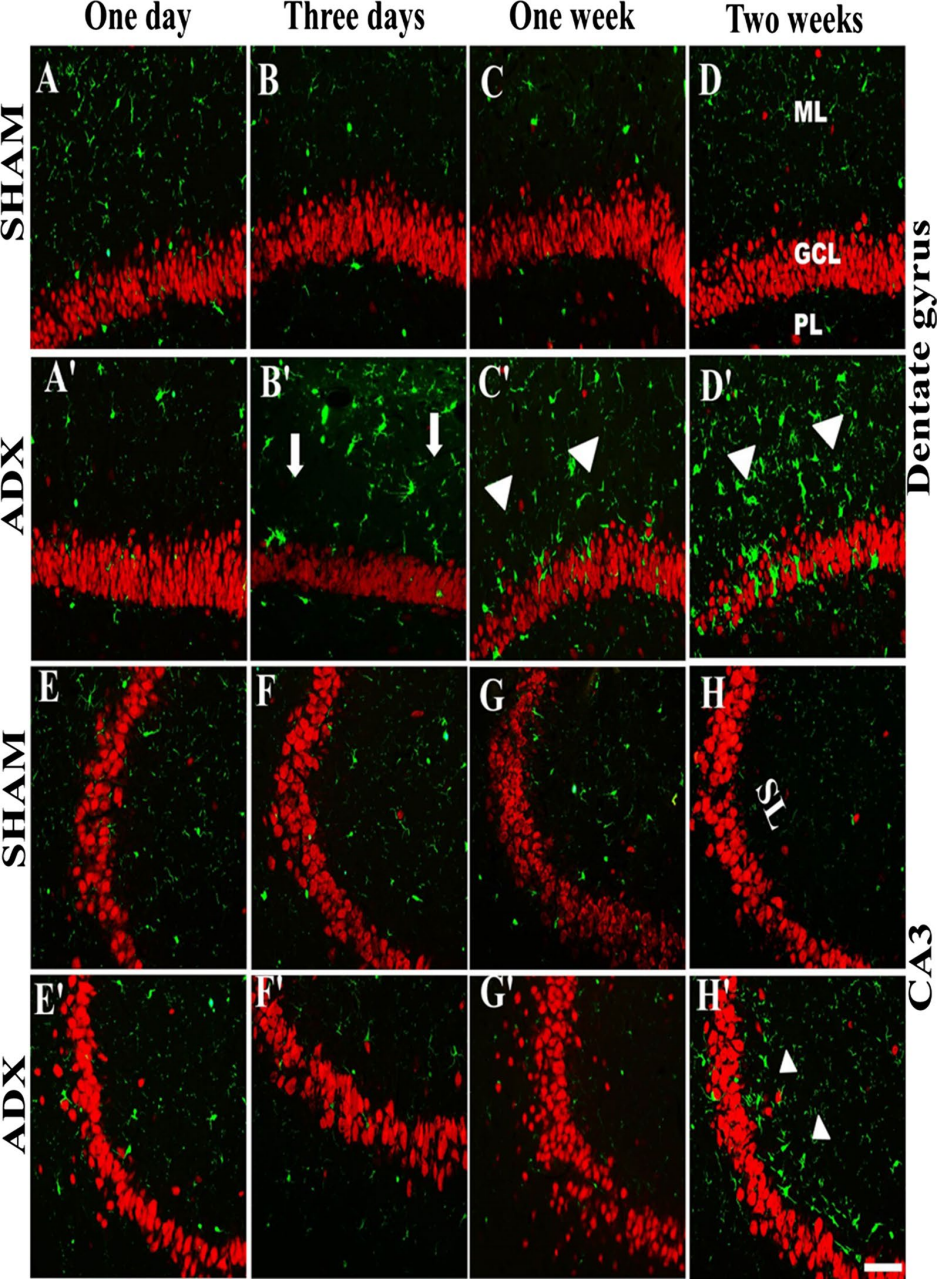
Images of coronal sections of the hippocampus stained with NeuN antibody (*red*) as a neuronal marker and Iba-1 antibody (*green*) as a microglia marker showing the progression of microgliosis over the course of time (1 day, 3 days, 1 week and 2 weeks) in the dentate gyrus and CA3 of the hippocampus of adrenalectomized rats compared to sham operated rats. Absence of microgliosis in the dentate gyrus (**A**, **B**, **C**, **D**) and CA3 (**E**, **F**, **G**, **H**) of sham operated rats during 1 day, 3 days, 1 week and 2 weeks. The adrenalectomized rats showed no signs of microgliosis in the dentate gyrus (**A**’) and CA3 (**E**’) after 1 day. However, a progression of microgliosis was seen in the dentate gyrus after 3 days (**B**’), 1 week (**C**’) and 2 weeks (**D**’). In comparison, the microgliosis in the CA3 of adrenalectomized rats did not appear until after 2 weeks as the Iba1 immunoreactivity after 1, 3 days and 1 week was not significantly different from that in sham operated rats. Molecular layer (ML), granule cell layer (GCL), polymorphous layers (PL) and (SL) stratum lucidum. *Scale bar* 50 µm

**Fig. 6:**
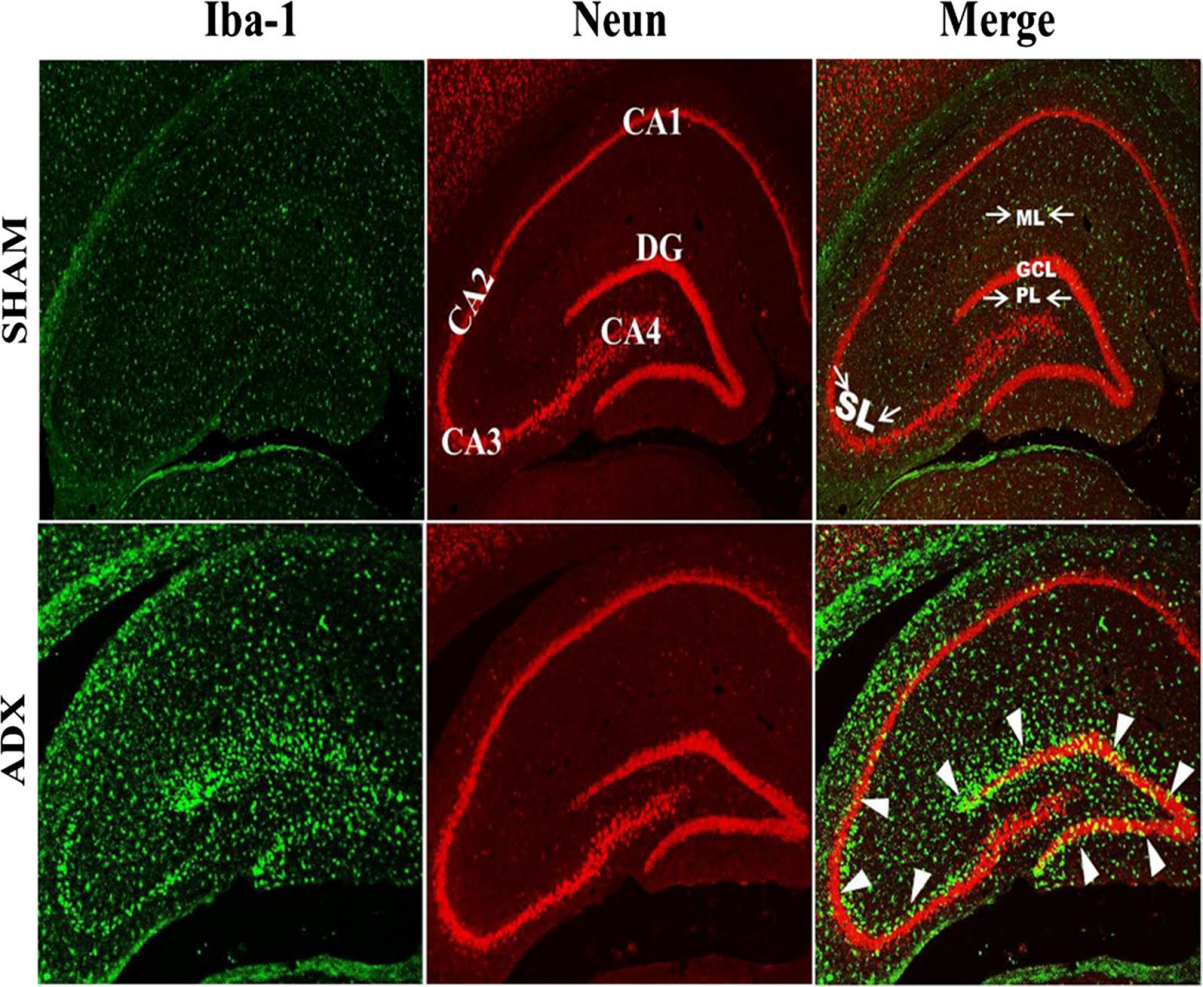
Representative images of coronal sections of the whole hippocampus stained with NeuN (*red*) and Iba-1 (*green*) antibodies showing microgliosis after 2 weeks of adrenalectomized rats compared to bilateral sham operated rats. Molecular layer (ML), granule cell layer (GCL), polymorphous layers (PL) and (SL) stratum lucidum. *Scale bar* 100 µm

Our immunoflurescent results were seen in different animals, in a consistent way and in the same pattern. Regarding microgliosis in the hippocampus, the fluorescent double immunostaining of neurons and microglia of brain sections after 4 h (results not shown) and 1 day of adrenalectomy did not show activated microglia in adrenalectomized and sham operated groups (Fig. 5A, A’). However, after 3 days we observed activated microglia in the dorsal blade of the dentate gyrus of adrenalectomized rats while no sign of activation was seen in the sham operated rats (Fig. 5B, B’). 1 week following the adrenal gland removal, activated microglia were seen in the whole dorsal blade of the dentate gyrus indicating a progression of microgliosis (Fig. 5C’). In contrast, no sign of activated microglia were observed in the hippocampus of sham operated rats (Fig. 5C). Fluorescent double immunostaining of neurons and microglia of brain sections after 2 weeks postoperatively revealed a strong Iba-1 immunoreactivity in the hippocampus of adrenalectomized rats most particularly in the dentate gyrus (Fig. 5D’) and stratum lucidum (SL) of CA3 (Fig. 5H’) areas compared to the corresponding regions in the sham operated rats (Fig. 5D, H). Iba-1 labeling exhibited a differential distribution throughout the dentate gyrus where it shows an intensive microgliosis in the molecular (ML) and granule cell layer (GCL) of the dorsal blade and predominately at the tip where the Neun staining shows apparent absence of neurons compared to the ventral blade 2 weeks post operatively (Fig. 6). Interestingly, at the same time point, we observed for the first time a strip of Iba-1 positive cells in the stratum lucidum layer of the CA3 area (Fig. 6) and (Fig. 5D’). No sign of microgliosis was seen in CA3 area of sham operated rats over the course of time (Fig. 5E, F, G, H).

**Fig. 7:**
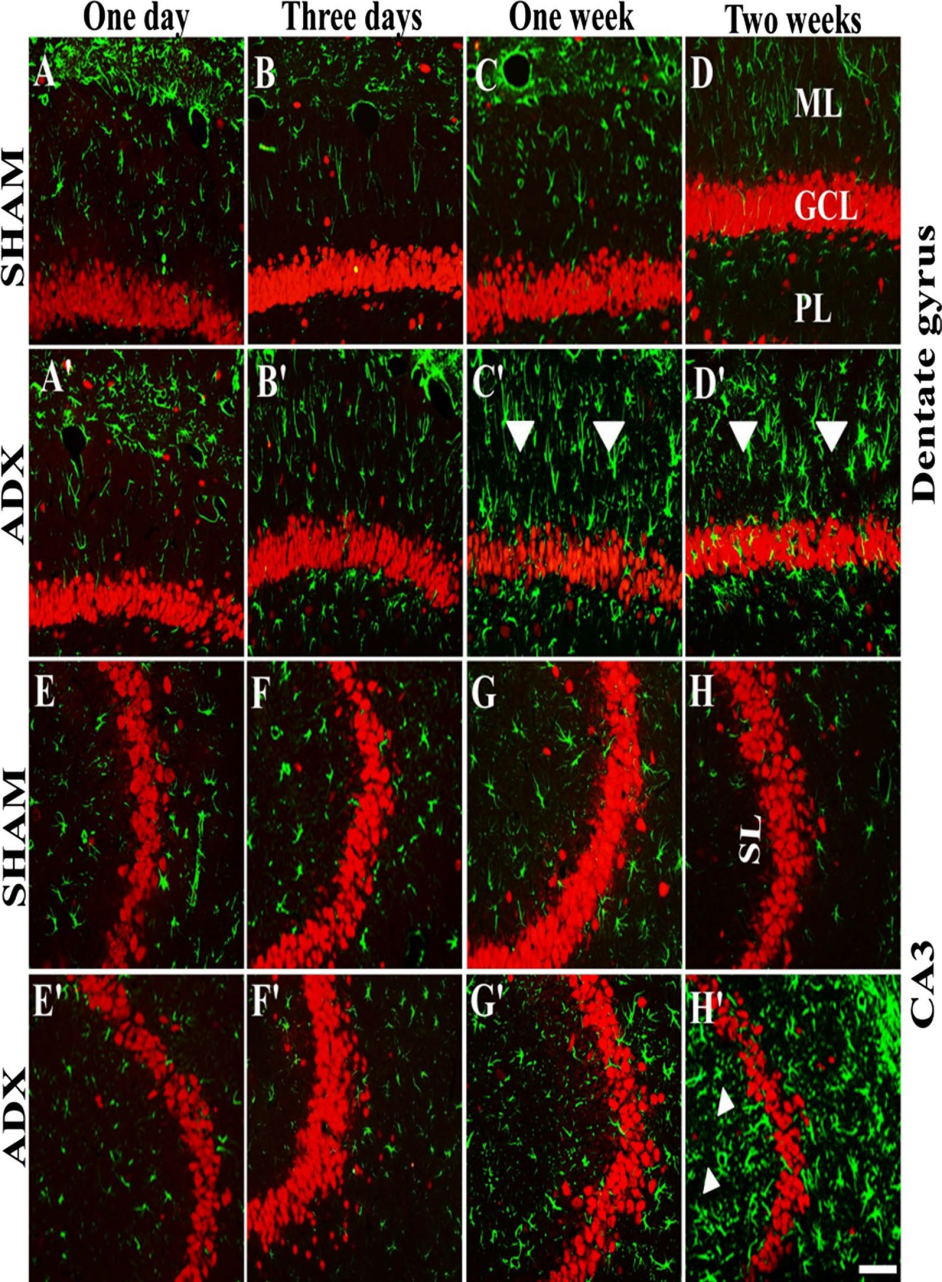
Images of coronal sections of the hippocampus stained with NeuN antibody (*red*) as a neuronal marker and GFAP antibody (*green*) as an astrocytes marker showing the progression of microgliosis over the course of time (1 day, 3 days, 1 week and 2 weeks) in the dentate gyrus and CA3 of the hippocampus of adrenalectomized rats compared to sham operated rats. Absence of astrogliosis was noticed in the dentate gyrus (**A**, **B**, **C**, **D**) and CA3 (**E**, **F**, **G**, **H**) of sham operated rats during the different time points (1 day, 3 days, 1 week and 2 weeks). 1 day and 3 days after adrenalectomy the rats showed no signs of astrogliosis in the dentate gyrus (**A**’, **B**’) and CA3 (**E**’, **F**’) respectively. A progression of astrogliosis was seen in the dentate gyrus after 1 week (**C**’) and 2 weeks (**D**’) whilst no immunoreactivity was seen in CA3 after 1 week (**G**’). However appearance of astrogliosis in CA3 was seen after 2 weeks (**D**’). Molecular layer (ML), granule cell layer (GCL), polymorphous layers (PL) and (SL) stratum lucidum. *Scale bar* 50 µm

In order to examine the effect of adrenal gland removal on astrocyte immunoreactivity in the hippocampus over a course of time (4 h, 24 h, 3 days, 1 week and 2 weeks), a double immunofluorescent labeling of neurons (Neun) and astrocytes (GFAP) was performed. After 4 h (results not shown), 1 and 3 days of adrenalectomy, we did not observe an apparent difference in the GFAP immunoreactivity in the hippocampii of adrenalectomized and sham groups (Fig. 7A, A’, B, B’). However, 1 week after adrenalectomy, activated astrocytes were seen in the dentate gyrus (Fig. 7C’) whilst no difference in the GFAP immunoreactivity was observed in sham operated rats (Fig. 7C).

**Fig. 8:**
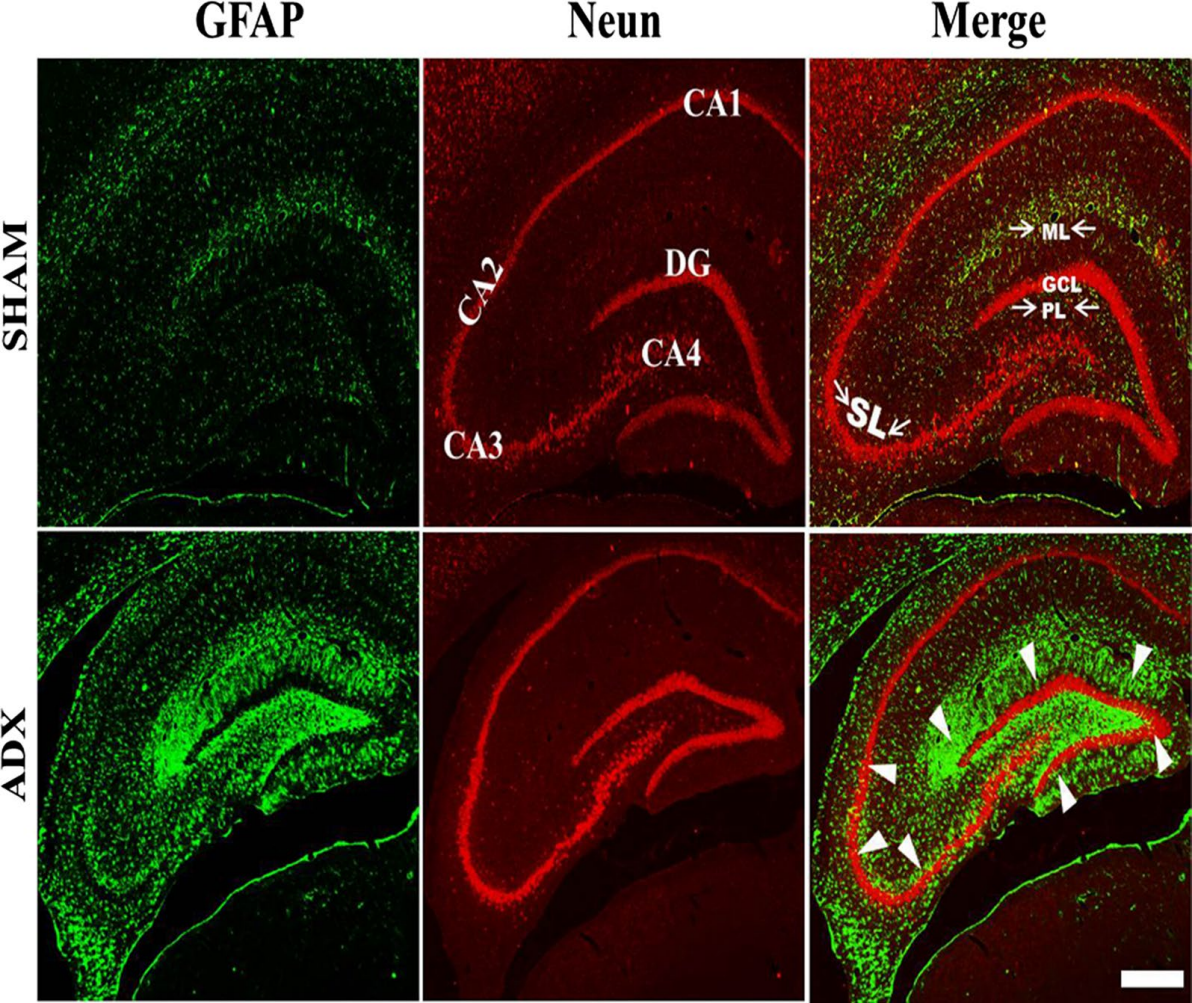
Representative images of coronal sections of the whole hippocampus stained with NeuN (*red*) and GFAP (*green*) antibodies showing astrogliosis after 2 weeks of adrenalectomized rats compared to bilateral sham operated rats. Molecular layer (ML), granule cell layer (GCL), polymorphous layers (PL) and (SL) stratum lucidum. *Scale bar* 100 µm

An intense GFAP immunoreactivity was observed 2 weeks after adrenalectomy, throughout the hippocampus most particularly in the dentate gyrus (Fig. 8) where the astrocytes undergo morphological changes and appeared hypertrophic and showed intense GFAP immunoreactivity (Fig. 7D’). The GFAP immuno-labeling exhibited extensive astrogliosis in the molecular and polymorphous layers (PL) of the dentate gyrus; however less intense astrogliosis was seen in the granular layer (Fig. 8). The stratum lucidum of CA3 area of adrenalectomized rats showed an increase in GFAP immunoreactivity (Fig. 7H’). Moreover, GFAP immunostaining also showed extensive astrogliosis in the CA4 but not in the CA1 area of the adrenalectomized rats (Fig. 8). The dentate gyrus (Fig. 7A, B, C, D), CA3 (Fig. 7E, F, G, H), CA4, CA2 and CA1 areas in the hippocampi of bilateral sham operated rats did not show astrogliosis (Fig. 8).

**Fig. 9:**
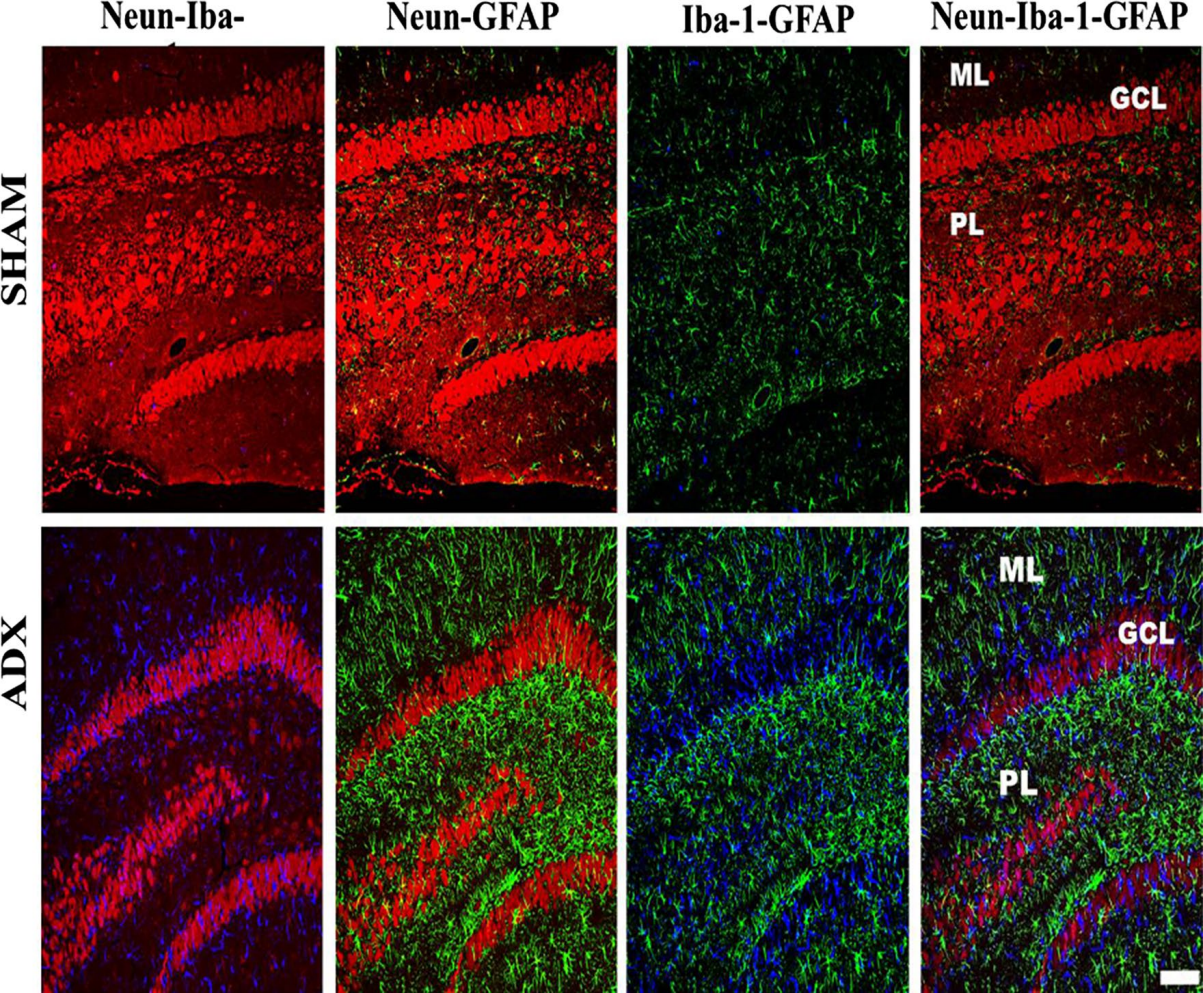
Representative confocal images of sections stained with Neun (*red*), Iba1 (*blue*) and GFAP (*green*) showing the localization of activated microglia and astroglia after 2 weeks throughout the dentate gyrus of the hippocampus of adrenalectomized rats compared to sham operated rats. Molecular layer (ML), granule cell layer (GCL), polymorphous layers (PL). *Scale bar* 50 µm

In order to investigate the localization of the activated microglia and astrocytes simultaneously after 2 weeks of adrenalectomy a triple immunostaining, of neurons (Neun), microglia (Iba-1),and astrocytes (GFAP) was performed and the sections were examined with a confocal microscope. We observed co-localization of the microglia and astrocytes in different layers of the dentate gyrus (Fig. 9).

**Fig. 10:**
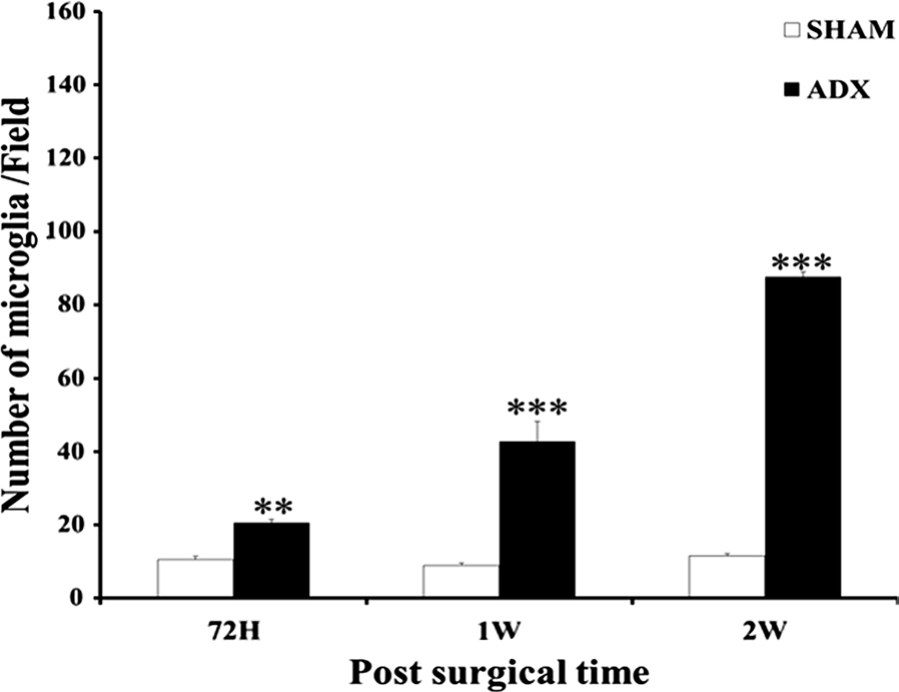
*Bar* graphs showing the comparison between the number of Iba-1 positive cells in the hippocampus of adrenalectomized and sham operated rats. The number of microglia was significantly and progressively increased in adrenalectomized rats 3 days, 1 week and 2 weeks postoperatively. **P < 0.01; ***P < 0.001. Data are expressed as mean (±SEM)

**Fig. 11:**
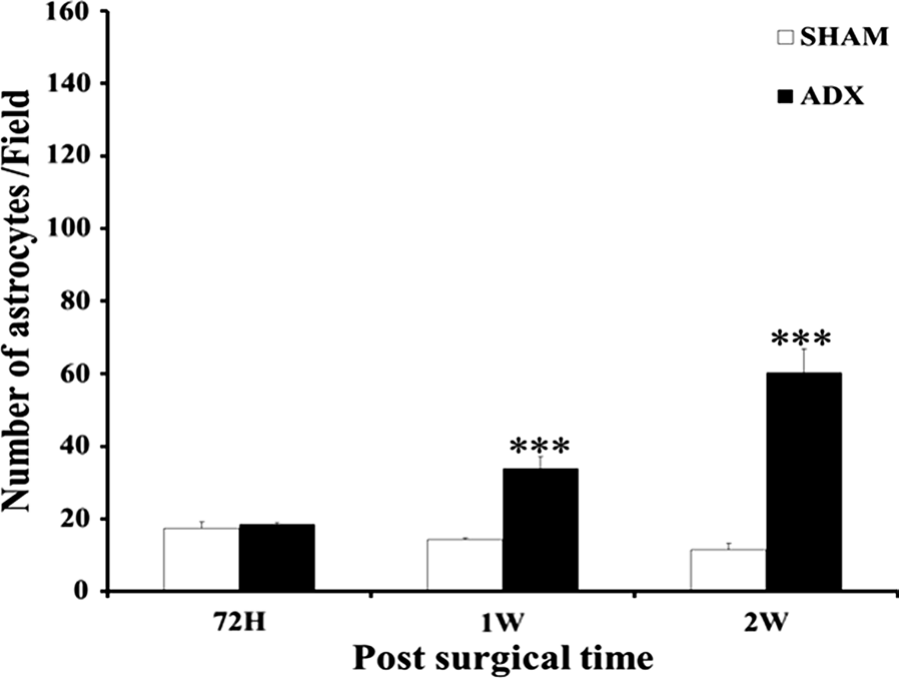
*Bar* graphs showing the comparison between the number of GFAP positive cells in the hippocampus of adrenalectomized and sham operated rats. No difference in the number of astrocytes revealed after 3 days but significantly increased 1 and 2 weeks following adrenalectomy. ***P < 0.001. Data are expressed as mean (±SEM)

The quantitative analysis showed that the number of Iba-1-positive microglia in the hippocampus of ADX rats was significantly higher in days 3 (P < 0.01), 7 (P < 0.001) and 14 days (P < 0.001) compared to the sham operated rats. The results showed progressive increase in the number of microglia in the hippocampus from day three with a further increase one and 2 weeks after adrenalectomy (Fig. 10). On the other hand, there was no significant difference between the number of GFAP-positive astrocytes in the hippocampus of ADX and sham operated rats 3 days following adrenalectomy. However, a significant increase (P < 0.001) in the number of astrocytes was observed one and 2 weeks after adrenalectomy in the ADX compared to sham operated rats (Fig. 11).

### Induction of oxidative stress state in the hippocampus after 2 weeks of adrenalectomy

**Fig. 12:**
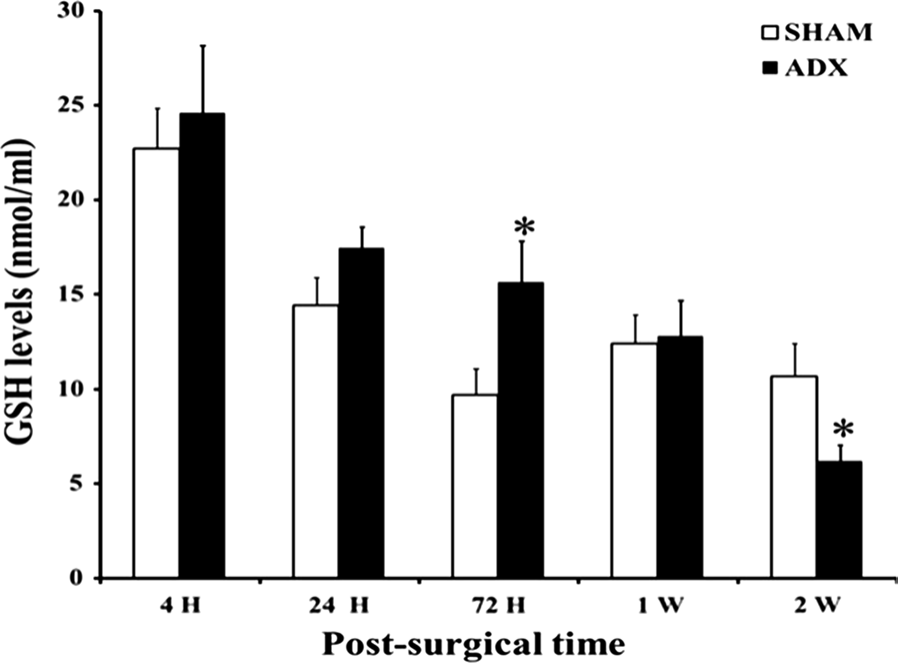
*Bar* graphs showing GSH levels in the hippocampus of adrenalectomized and Sham operated rats. Levels of GSH were measured by colorimetric assay over course of time (4 h, 24 h, 3 days, 1 week and 2 weeks) in adrenalectomized compared to sham operated rats.*P < 0.05. Data are expressed as mean (±SEM)

Reduced glutathione (GSH) is the major reducing antioxidant by serving as an electron donor and detoxification of free radicals in the cell. As shown in (Fig. 12) the evaluation of the levels of this antioxidant component in the hippocampal homogenates over the course of time (4 h, 24 h, 3 days, 1 week and 2 weeks) in bilateral adrenalectomized rats compared to sham operated indicated no significant increase in the first day following surgery, however, at day three a significant increase (P < 0.05) in the levels of GSH was observed in adrenalectomized rats compared to the sham. At 1 week postoperatively, no significant difference in the levels of GSH was seen between the two groups, however, 2 weeks later we noticed a significant decrease (P < 0.05) in GSH levels in adrenalectomized rats.

**Fig. 13:**
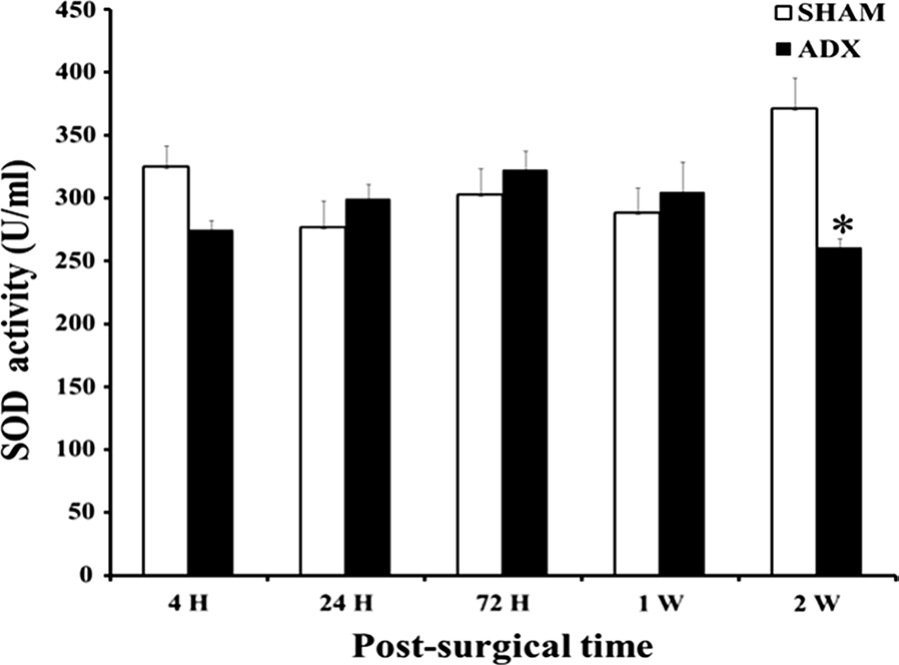
*Bar* graphs showing SOD activity in hippocampus of adrenalectomized and sham operated rats. Levels of SOD activity were measured by colorimetric assay over the course of time (4 h, 24 h, 3 days, 1 week and 2 weeks) in adrenalectomized compared to sham operated rats.*P < 0.05. Data are expressed as mean (±SEM)

The measurement of the antioxidant enzyme, superoxide dismutase (SOD) activity was performed by a colorimetric assay in the hippocampal homogenates of adrenalectomized compared to sham operated rats. Our results indicated no significant changes in SOD activity in the hippocampal homogenates of adrenalectomized compared to sham operated rats after 4 h, 24 h, 72 h and 1 week of adrenalectomy. However, 2 weeks after surgery a significant decrease in the enzyme activity (P < 0.01) was revealed in the hippocampus of adrenalectomized compared to sham operated rats (Fig. 13).

**Fig. 14:**
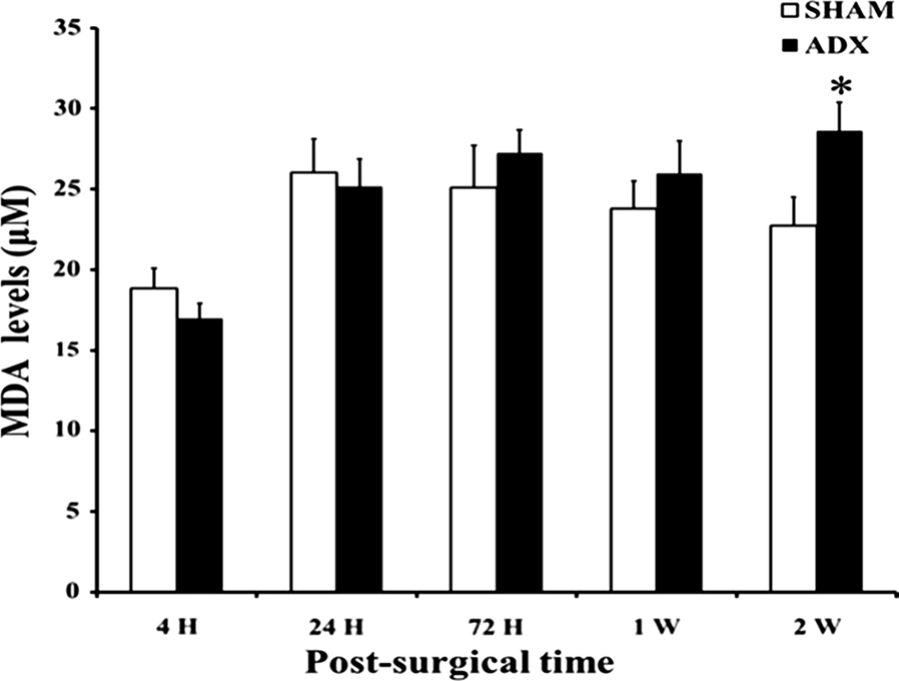
*Bar* graphs showing MDA levels in the hippocampus of adrenalectomized and Sham operated rats. Levels of MDA were measured by colorimetric assay over course of time (4 h, 24 h, 3 days, 1 week and 2 weeks) in adrenalectomized rats compared to sham operated rats. *P < 0.05. Data are expressed as mean (±SEM)

Lipid peroxidation is one of the most important manifestations of the imbalance between the antioxidants and the oxidants which leads to oxidative stress. We examined the occurrence of lipid peroxidation in the hippocampus by the measurement of the levels of malondialdehyde (MDA), the lipid peroxidation by-product. No statistical differences in the levels of MDA until 2 weeks after surgery when a significant increase (P < 0.05) in MDA levels was seen in adrenalectomized rats compared to sham operated rats (Fig. 14).

## Discussion

The aim of this study was to investigate the effects of short-term bilateral adrenalectomy on the levels of pro-inflammatory cytokines IL-1β, IL-6 and TNF-α, the response of microglia and astrocytes to neuronal cell death as well as oxidative stress markers GSH, SOD and MDA over the course of time (4 h, 24 h, 3 days, 1 week and 2 weeks) in the hippocampus.

Our results showed transient elevation of IL-1β and IL-6 levels which lasts for 3 days in the hippocampus of adrenalectomized compared to sham operated rats. 1 week after adrenalectomy, the elevation of these cytokines dropped to sham levels. Both the sham operated and adrenalectomized rats showed significant elevated IL-1β and IL-6 levels compared to the naïve rats. The significant difference between the naïve and sham operated animals might be attributed to the surgical stress. However, the significant difference between the sham and adrenalectomized rats can only be attributed to the effect of adrenalectomy. We noted significant difference in the hippocampal levels of TNF-α between adrenalectomized and sham operated rats only at 4 h after adrenalectomy.

The occurrence of neuronal cell death in the hippocampus following adrenalectomy was confirmed by Fluoro-Jade B stain. Our results showed a time dependent increase in degenerated neurons in the dorsal blade of the dentate gyrus from 3 days to 1 week after adrenalectomy. 2 weeks after adrenalectomy we observed a progression of cell death throughout the dorsal blade of the dentate gyrus. These findings are in line with those of Spanswick et al. [59] who reported, degenerated neurons throughout the granular layer of the dentate gyrus 2 weeks after adrenalectomy using Fluoro-Jade B staining. After 2 weeks we observed little cell death in the ventral blade. Our findings indicate that adrenalectomy affects differentially the dorsal and ventral blades of the dentate gyrus and suggest that the granule cells of the dorsal blade of the dentate gyrus are more vulnerable to adrenalectomy. In line with our findings, several investigators have observed that the earliest cell death occurs at least after 3 days of adrenalectomy [7, 11, 59–61]. However, few investigators reported cell death on day two after adrenalectomy [7, 12, 62].

It is interesting to note that the early elevation of the pro-inflammatory cytokines IL-1β, IL-6 and TNF-α was seen prior to neuronal death. Taken together our findings suggest that cell death was preceded by an increase of these pro-inflammatory cytokines. Similar findings have been reported in animal models of ischemia [19, 20], surgical injury [21], TMT (*T*rimethyltin) [22] KA (Kainic acid) [23] and cis-2,4-methanoglutamate (MGlu) [24] where an early increase of different pro-inflammatory cytokines preceded neuronal damage.

Two studies reported the direct involvement of IL-1β in neurodegeneration. Rothwell [19] reported the injection of low doses of IL-1β into the cerebral ventricles or brain parenchyma of mice or rats exposed to neurotoxins markedly exacerbates the damage. The second study showed the antagonism of IL-1β by co-infusion of IL-1β receptor antagonist with the neurotoxin N-Methyl-D-aspartate (NMDA) inhibits brain damage induced by such excitotoxin [63, 64]. Moreover, IL-1β plays a crucial role in the exacerbation of acute neurodegeneration caused by ischemia, head trauma and stroke. In addition, it is implicated in the pathology of multiple sclerosis, Alzheimer’s disease and other CNS chronic diseases [24, 65–67].

IL-6 levels were found to be elevated in the serum and CSF of MS patients, locally around MS lesions [68, 69] and in experimental auto-immune encephalitis (EAE) animal models [70]. IL-6 expression was significantly increased in the acute phase of cerebral ischemia [44]. In AD patients, IL-6 is further increased locally around amyloid plaques and in the CSF [34, 71].Taken together our findings suggest that after adrenalectomy IL-1β, IL-6 and TNF-α might contribute to the initiation of the biological cascade responsible for subsequent hippocampal neuronal cell death.

The activation of microglia is one of the major signs of neurodegeneration [71]. Similar to Gould et al. [60], our results showed cell death starts in the dorsal blade of the dentate gyrus 3 days after adrenalectomy. Activated microglia were observed in the molecular and granular layers of the dorsal blade of the dentate gyrus 3 days after adrenalectomy. 1 week after adrenalectomy as the cell death progresses in the dorsal blade the number of activated microglia are increased. 2 weeks after adrenalectomy we showed that the whole dentate gyrus was invaded by activated microglia. In line with these findings, quantitative analysis showed a progressive increase in the number of microglia in the hippocampus from day three with a further increase 1 and 2 weeks after adrenalectomy. Jaarsma et al. [62], using silver impregnation staining, observed argyrophilia in the somata and dendrites of the granular and molecular layers respectively and in the mossy fiber terminal zone. Regarding the cornu amonis, 2 weeks after adrenalectomy we observed invasion of the CA4 by activated microglia. In contrast, no sign of microgliosis was seen in the pyramidal cells of CA3, CA2 and CA1. However, we noticed activated microglia along the stratum lucidum of the CA3. Our current study showed a strong correlation between cell death and microglia activation over the course of time. Taken together our observations suggest a correlation between microgliosis and cell death.

It is well known that brain injury is associated with increased astrocytes reactivity [37]. Astrocytes react profoundly to brain damage or disease by increasing both their number and size, a process referred to as reactive astrogliosis/astrocytosis which represents a remarkably homotypic response to CNS injuries [72]. In this study, activation of astrocytes was observed throughout the dentate gyrus 1 week after adrenalectomy. However, Krugers et al. [14] found activation of astrocytes earlier, on the third day after adrenalectomy. Although we could not see activated astrocytes at day three, it is possible that the activation of astrocytes takes place between day 3 and 7 after adrenalectomy. The observed astrogliosis in our study was predominantly in the molecular and polymorphous layers of the dentate gyrus whilst no apparent astrogliosis was seen in other areas of the hippocampus. Our findings are in line with those findings which showed astrocytes invasion of the dentate gyrus after TMT injection. TMT is a selective neurotoxin of granule cells [37]. In addition to the activation of astrocytes after 1 week in the DG our results showed progressive activation of astrocytes in the CA4 and CA3 of the hippocampus 2 weeks after adrenalectomy. In line with these findings, quantitative analysis showed a significant increase in the number of astrocytes in the hippocampus from 1 week until 2 weeks after adrenalectomy.

Astrocytes are known to communicate with microglia, and such interaction is very important in pathological conditions [73, 74]. The progressive degeneration of granule cells correlated well with the activation of microglia and astroglia in the dentate gyrus over the course of 2 weeks.

Our results showed microglia activation on the third day followed by astrocytes activation on the seventh day after adrenalectomy. These findings are in line with what has been found in different neurodegenerative models where microglia were found to be the first cells to respond to neuronal damage followed by activation of astrocytes [75–78]. Taken together these findings suggest a cross talk between the two cell populations during neuronal damage and consider microglia as driving force in astroglia recruitment.

Oxidative stress occurs when there is an overproduction, or accumulation of reactive oxygen species (ROS) in conjunction with reduced antioxidant capacity within the cell [79]. As a response to neuronal death, the quiescent microglia became functionally active and upregulate enzymes such as inducible nitric oxide synthase (iNOS) leading to an imbalance between free radicals production and the antioxidant defenses [80].

Our findings showed that a state of oxidative stress manifested in the decrease of reduced glutathione (GSH) 2 weeks after adrenalectomy. In addition, our study showed a significant decrease in the activity of the superoxide dismutase. In contrast to the decrease of the antioxidant defenses, malondialdehyde (MDA) the indicator of the plasma membrane disruption was significantly increased. This imbalance, observed 2 weeks after adrenalectomy, reflects a state of oxidative stress which possibly resulted from the activated glial cells. It has been found after 1 week of adrenalectomy an overexpression of iNOS in activated microglia revealing these cells as source of the free radical in this model [16]. Taken together our findings are consistent with the literature showing that several neurodegenerative paradigms revealed the association between neuroinflammation accompanied by reactive gliosis and a state of oxidative stress [81].

The mechanisms underlying cell degeneration after bilateral adrenalectomy are not clear. We have previously suggested the possibility that adrenocortical hormones might have direct effects on the survival of hippocampal neurons. Hippocampal neurons might die when the mineralocorticoids (Type I) and/or glucocorticoid (Type II) receptors are not occupied by adrenocortical hormones [18, 82]. Subsequently, Hu et al. [83] reported that granule cell death after adrenalectomy was necessarily accompanied by the disappearance of glucocorticoid receptor immunoreactivity in the granule cell layer. We have previously also suggested that the loss of adrenocortical hormones after adrenalectomy may cease to stimulate a factor(s), in the hippocampus or other tissues, that is (are) necessary for the survival of hippocampal neurons [18, 82]. It could be speculated that in several neurodegenerative diseases like Alzheimer’s disease, chronic adrenal hormones increase and/or their receptors’ decrease may result in inflammatory mechanisms which lead to hippocampal neurodegeneration [84–88].

## Conclusion

Our study showed that short-term bilateral adrenalectomy leads to an early increase in pro-inflammatory cytokines followed by neurodegeneration and activation of glial cells as well as oxidative stress. Taking these findings together it could be suggested that the early inflammatory components might contribute to the initiation of the biological cascade responsible for subsequent hippocampal neuronal cell death in the current neurodegenerative animal model. These findings suggest that inflammatory mechanisms precede neurodegeneration and glial activation.

## Abbreviations

AD: Alzheimer’s disease
ALS: amyotrophic lateral sclerosis
BDNF: brain derived growth factor
EAE: experimental auto-immune encephalitis
EIA: enzyme immunoassay
DTNB: 5,5′-dithio-bis-(2-nitrobenzoic acid)
DG: dentate gyrus
GFAP: glial fibrillary acidic protein
GSH: reduced glutathione
Iba1: ionized calcium-binding adaptor molecule 1
IL-1β: intrleukin-1
IL-6: intrleukin-6
iNOS: inducible nitric oxide synthase
IGF-1: insulin-like growth factor
MDA: malondialdehyde
MPTP: 1-methyl-4-phenyl-1,2,3,6-tetrahydropyridine
MS: multiple sclerosis
NeuN: neuronal nuclear antigen
NGF: nerve growth factor
NO: nitric oxide
NT: neurotrophin
PBS: phosphate-buffered saline
PD: Parkinson’s disease
ROS: reactive oxygen species
SPSS: statistical package for the social sciences
SOD: superoxide dismutase
TMT: trimethyl tin
TNF-α: tumor necrosis factor
